# Predictors of mortality and clinical outcomes following implantable cardioverter‐defibrillator therapy in elderly patients: A retrospective single‐center cohort study

**DOI:** 10.1002/hsr2.1432

**Published:** 2023-07-22

**Authors:** Alireza Malekrah, Akbar Shafiee, Amirhossein Heidari, Ali Vasheghani‐Farahani, Ali Bozorgi, Saeed Sadeghian, Ahmad Yaminisharif

**Affiliations:** ^1^ Department of Electrophysiology, Tehran Heart Center, Cardiovascular Diseases Research Institute Tehran University of Medical Sciences Tehran Iran; ^2^ Cardiovascular Research Center Mazandaran University of Medical Science Sari Iran; ^3^ Department of Cardiovascular Research, Tehran Heart Center, Cardiovascular Diseases Research Institute Tehran University of Medical Sciences Tehran Iran; ^4^ Cardiac Primary Prevention Research Center, Cardiovascular Diseases Research Institute Tehran University of Medical Sciences Tehran Iran; ^5^ Faculty of Medicine, Tehran Medical Sciences Islamic Azad University Tehran Iran

**Keywords:** elderly, implantable cardioverter‐defibrillator, life expectancy, survival

## Abstract

**Background and aims:**

Implantable cardioverter‐defibrillators (ICDs) are frequently used to prevent sudden cardiac death in patients with high‐risk arrhythmias. However, the use of ICD therapy in elderly patients beyond the predicted age of life expectancy is still controversial. We aimed to evaluate the predictors of mortality and clinical outcomes following ICD implantation in elderly patients.

**Methods:**

We conducted a retrospective analysis of 145 elderly patients aged 72 years and older who received ICD implantation between January 2010 and August 2015. We collected and analyzed baseline data, including clinical, demographic, and medical history, the reason for ICD therapy, procedural data, and echocardiography results. Follow‐up data included the development of complications and mortality. The predictors of mortality were identified using the univariate and multivariable Cox regression models.

**Results:**

During the median follow‐up duration of 30.5 [18.0–48.0] months, 141 cases completed follow‐up (mean age = 76.0 ± 3.7 years). Forty‐four patients experienced at least one episode of ICD therapy. Inappropriate shock, recurrent shock, and device‐related infection were the most frequent complications observed in our study. Of the 145 patients, 42 died during the follow‐up period, with an average survival time of 22.4 months after ICD implantation. Among these patients, 11 received ICD for primary prevention, and 31 received it for secondary prevention. Cardiovascular problems were the leading cause of death. We found that a low baseline ejection fraction (EF) was an independent predictor of mortality (hazard ratio = 0.93, 95% confidence interval: 0.90–0.98; *p* = 0.008).

**Conclusion:**

Our study suggests that ICD therapy is a valuable treatment option for elderly patients beyond their predicted age of life expectancy. The study highlights the importance of baseline EF as a significant predictor of mortality in these patients.

## 1 INTRODUCTION

In recent decades, the utilization of implantable cardioverter‐defibrillators (ICDs) has steadily risen globally.^1^ This trend reflects the increasing recognition of the effectiveness of ICDs in managing a wide range of cardiac conditions, including arrhythmias and heart failure.^2^ ICDs have emerged as a standard therapy for preventing sudden cardiac death, particularly in cases of life‐threatening ventricular arrhythmias.^3^ With a proven efficacy and safety track record, ICDs have become the most effective preventive strategy for managing cardiac conditions with a high risk of sudden death.^4^, ^5^ Despite the proven efficacy of ICDs, it remains unclear whether ICD therapy provides similar benefits for primary prevention in older individuals.

As individuals age, they often develop multiple chronic diseases, complicating medical decision‐making.^6^ This is particularly challenging when treating elderly patients who have already exceeded their predicted life expectancy. The selection of an appropriate treatment plan becomes increasingly complex due to the coexistence of multiple chronic conditions, which can significantly impact their survival.^7^ Therefore, addressing this issue is essential for effectively managing elderly patients with multiple chronic diseases. According to current guidelines, ICD therapy is recommended for patients with end‐stage heart failure or those with a life expectancy of at least 1–2 years.^3^, ^8^

In addition to considering comorbidities, the quality of life of a patient is also an essential factor to consider when making decisions about ICD therapy.^9^ While age is a critical factor in determining the appropriate treatment plan for elderly patients, it should not be the only criterion for deciding on ICD therapy.^10^, ^11^ Regrettably, there is a scarcity of data regarding cardiovascular interventions in older adults.^12^, ^13^ As the population of elderly individuals continues to expand, there is an urgent need for further research to address the knowledge gaps and inform the cardiovascular care of this growing population.

Therefore, we aimed to investigate the predictors of mortality and clinical outcomes of elderly patients who received ICD therapy at our center.

## 2 METHODS

### 2.1 Study design

This retrospective cohort study evaluated a comprehensive data review from 1198 patients who received ICD therapy at our center between January 2010 and August 2015. For this study, we included older adults who underwent ICD implantation for primary or secondary prevention and followed up for 5 years above the predicted life expectancy in Iran (72 years for men and 74 years for women in 2010 ^14^) during this period. To ensure the quality and accuracy all data were retrieved from the Tehran Heart Center databank and completed with the patient's medical records during their follow‐up visits. Additionally, we excluded cases with incomplete data or missed follow‐ups.

In accordance with our hospital's routine, all patients provided written informed consent upon admission, granting permission for their clinical data to be used anonymously for research purposes. Tehran University of Medical Sciences and the Cardiology Department Research Committee approved the study protocol. (Filing code: 782, Date: 21/12/2015). This study conforms to the ethical principles outlined in the Declaration of Helsinki and its updates.

### 2.2 Data collection and follow‐up

We comprehensively evaluated patient data, including baseline information such as demographic and clinical characteristics, medical history, procedural data, and echocardiography results. A number of clinical and demographic data were considered as baseline characteristics, including age, gender, hypertension, diabetes mellitus, history of coronary artery disease and cardiovascular disease, history of the coronary artery bypass graft, cardiomyopathy, chronic obstructive pulmonary disease, chronic kidney disease, and other comorbidities such as hepatic failure, Alzheimer and dialysis history, lead fracture, and malignancy as well as the type of ICDs device and baseline ejection fraction (EF). It should be clarified that baseline data was obtained before ICD implantation.

After implantation, patients visited our pacemaker clinic every 6 months, and we retrieved their follow‐up data from hospital records to ensure standardized device programming and ongoing monitoring. In cases where we were unable to obtain postoperative clinical records, we contacted patients via telephone to assess their vital status. All‐cause mortality was the primary endpoint of our study. In addition, we will also investigate the survival rates, cost, and benefit of ICD therapy as secondary endpoints.

### 2.3 Statistical analysis

Categorical variables were expressed as frequencies and percentages, and the Chi‐square test was used to compare the dead and alive patients. Continuous variables were presented as mean ± standard deviation and were compared between the study groups using the Student's *t*‐test after ensuring the normal distribution of the variable. Due to non‐normal distribution, the follow‐up period was reported as median [Interquartile range]. Univariate Cox regression analysis was performed to determine the predictors of mortality. The independent predictors of survival were identified by multivariate Cox regression analysis by including variables with a *p*‐value < 0.200 in the univariate analysis. Data analyses were performed using SPSS version 23.0 (IBM) software. A *p*‐value less than 0.05 was considered statistically significant for all tests.

## 3 RESULTS

### 3.1 Clinical characteristics

From January 2010 to August 2015, 1198 patients underwent ICD implantation in our center. Based on our inclusion criteria, 145 patients were older than 72 years, and four were excluded due to incomplete follow‐up data. Finally, 141 patients were included in this analysis. The mean age of the participants at the time of implantation was 76.0 ± 3.7 years; 29 (20.5%) individuals were ≥80 years old, and 109 (77.3%) were men. Moreover, the mean implant time for all cases was 89.8 ± 2.1. In addition, 107 (75.8%) patients received an ICD, and 34 (24.1%) cardiac resynchronization therapy device (CRT‐D). Thirty‐five patients received the device for primary prevention, with the main indication in most patients being ventricular tachycardia. The baseline characteristics of the participants are entirely depicted in Table 1.

**Table 1:** Study population baseline, clinical, and follow‐up characteristics in both alive and dead groups.

Characteristic	Total (*n* = 141)	Alive (*n* = 95)	Dead (*n* = 42)	*p*‐Value*
Age, mean ± SD, y	76.0 ± 3.7	75.9 ± 8.0	76.9 ± 3.0	0.434
Male sex, *n* (%)	109 (76.9)	78 (82.1)	31 (73.8)	0.267
EF, mean ± SD, *n* (%)	30.50 ± 20.0	32.3 ± 20.0	28.17 ± 15.0	0.226
Device type, *n* (%)				0.934
ICD	105 (76.6)	73 (76.8)	32 (76.2)	
CRT	32 (23.4)	22 (23.2)	10 (23.8)	
Shock, *n* (%) Yes	44 (33.6)	31 (33.3)	13 (34.2)	0.923
Shock type, *n* (%)				0.233
Appropriate	31 (77.5)	20 (71.4)	11 (91.7)	
Inappropriate	9 (22.5)	8 (28.6)	1 (8.3)	
Indication, *n* (%)				0.832
Primary	35 (24.8)	24 (25.2)	11 (26.1)	
Secondary	100 (70.9)	70 (73.6)	30 (71.4)	
Change device	6 (4.3)	1 (1.6)	1 (2.4)	
Alzheimer, *n* (%)	1 (0.7)	0 (0.0)	1 (2.4)	0.307
CABG, *n* (%)	30 (21.9)	22 (23.2)	8 (19)	0.592
CAD, *n* (%)	61 (44.5)	40 (42.1)	21 (50.0)	0.391
Cardiomyopathy, *n* (%)	6 (6.3)	6 (4.4)	0 (0)	0.177
CKD, *n* (%)	7 (5.1)	5 (5.3)	2 (4.8)	0.999
COPD, *n* (%)	5 (3.6)	2 (2.1)	3 (7.1)	0.168
CVA. *n* (%)	8 (5.8)	4 (4.2)	4 (9.5)	0.249
CVD, *n* (%)	4 (2.9)	2 (2.1)	2 (4.8)	0.586
Dialysis, *n* (%)	2 (1.5)	0 (0)	2 (4.8)	0.920
DM, *n* (%)	8 (5.8)	8 (8.4)	0 (0)	0.106
Hepatic failure, *n* (%)	2 (1.5)	0 (0)	2 (4.8)	0.92
Lead Fracture, *n* (%)	2 (1.5)	2 (2.1)	0 (0)	0.999
Malignancy, *n* (%)	3 (2.2)	1 (1.1)	2 (4.8)	0.222
Recurrent shock, *n* (%)	4 (2.9)	4 (4.2)	0 (0)	0.312

Abbreviations: CABG, coronary artery bypass graft; CAD, coronary artery disease; CKD, chronic kidney disease; COPD, chronic obstructive pulmonary disease; CVA, cerebrovascular accident; CVD, cardiovascular disease; DM, diabetes mellitus; EF, ejection fraction.

* *p* < 0.05 was considered significant.

### 3.2 ICD therapy‐follow‐up and complications

The median follow‐up duration was 30.5 [18.0–48.0] months. During this period, and based on the device analysis report, 44 patients received at least one shock episode. Of these patients, 31 (77.5%) had an appropriate shock. In terms of ICD complications, nine patients (6.4%) had an inappropriate shock, one patient (0.7%) had a fractured lead, four patients (2.8%) suffered device‐related infections, and four (2.8%) patients had a recurrent shock.

### 3.3 Mortality details and survival rate

During the follow–up period, the overall survival rate was 67.3%, and 42 (29.7%) patients died. The leading causes of death were cardiac arrest in 11% (*n* = 5), sepsis or infection in 2.8% (*n* = 4), pulmonary edema in 7% (*n* = 3), myocardial infarction in 7.1% (*n* = 3), cerebrovascular accident in 9.5% (*n* = 4), gastrointestinal bleeding/disorder in 4% (*n* = 2), malignancy in 4.8% (*n* = 2), renal failure in 4.8% (*n* = 2), hepatic failure in 4.8% (*n* = 2), congestive heart failure in 2.4% (*n* = 1), accident in 2.4% (*n* = 1), and bowel obstruction in 2.4% (*n* = 1). In the remaining patients, the leading cause of death was unclear.

Of the deceased patients, 11 (31.4%) were in the primary prevention group, while 31 (29.2%) were in the secondary prevention group, and the difference was not statistically significant. The median survival benefit after ICD implantation was approximately 36.0 [18.0–48.0] months for the primary prevention group and 30.0 [18.0–48.0] months for the secondary prevention group, which was not statistically significant. The mean survival after ICD implantation was 18.0 [6.0–38.5] months for the deceased group and 36.0 [24.0–48.0] months for the group still alive at their last follow‐up.

### 3.4 Mortality predictors following ICD therapy

The univariable Cox regression analysis revealed that the most significant predictor of mortality after ICD implantation was a low baseline EF (*p* = 0.040) (Table 2). Notably, in the multivariable Cox regression model, the baseline EF remained the only independent predictor of mortality (hazard ratio [HR] = 0.93, 95% confidence interval [CI]: 0.89–0.98; *p* = 0.008) (Table 3).

**Table 2:** Univariate Cox‐regression analysis to predict survival of elderly patients following treatment with implantable cardioverter‐defibrillator.

	Univariable analysis		
Characteristic	HR	95% CI	*p*‐Value*
Age	1.02	0.92–1.12	0.721
Gender	0.61	0.31–1.23	0.173
Diabetes mellitus	0.44	0.0–7.51	0.234
CABG	0.66	0.3–1.44	0.307
Coronary artery disease	0.64	0.35–1.18	0.159
Cardiomyopathy	0.47	0.0–53.25	0.393
CKD	1.11	0.26–4.6	0.885
COPD	1.43	0.44–4.69	0.547
CVA	1.52	0.54–4.29	0.421
Ejection fraction	0.93	089–0.98	0.04
Device type	1.04	051–2.12	0.906
Shock	0.72	0.36–1.42	0.353
Indication (primary vs. secondary)	0.96	0.48–1.93	0.929

Abbreviations: CABG, coronary artery bypass graft; CI, confidence interval; CKD, chronic kidney disease; COPD, chronic obstructive pulmonary disease; CVA, cerebrovascular accident; HR, hazard ratio.

* *p* < 0.05 was considered significant.

**Table 3:** Multivariable cox‐regression analysis to predict survival of elderly patients following treatment with implantable cardioverter‐defibrillator.

	Multivariable analysis		
Characteristic	HR	95% CI	*p*‐Value*
Gender	0.66	0.25–1.79	0.423
Ejection fraction	0.93	0.89–0.98	0.008
Coronary artery disease	0.73	0.32–1.64	0.447

Abbreviations: CI, confidence interval; HR, hazard ratio.

* *p* < 0.05 was considered significant.

## 4 DISCUSSION

The main findings of the current study are that in elderly patients ( ≥ 72 years) undergoing ICD therapy, a low baseline EF is an independent predictor of mortality. During a median follow‐up of 30.5 months, 29.7% of the patients died. The survival benefit after ICD implantation was not statistically significant between primary and secondary prevention groups, and the mean survival was shorter in deceased patients than in those still alive at the last follow‐up. These findings highlight the importance of patient selection and monitoring in the elderly population undergoing ICD therapy.

Despite the rising number of elderly individuals in society and the growing need for ICD therapy, there is a dearth of reliable data regarding the effectiveness and consequences of this treatment. The current guideline acknowledges ICD therapy as a viable treatment option for the elderly.^15^ Moreover, it should be considered that older adults were excluded from the related clinical trials and that the definition of old age may differ from study to study. As proof of this claim, the average age of the studied population in previous major clinical trials, including Multicenter Automatic Defibrillator Implantation Trial II (MADIT‐II) and Sudden Cardiac Death in Heart Failure Trial (SCD‐Heft), were 64 and 60 years, respectively.^16^, ^17^ A recent multicenter study focusing on ICD therapy in the elderly reported a median age of 78.6.^7^

Several studies have been conducted to evaluate the benefits of ICD therapy in elderly patients, but the definition of the elderly age group has not been clarified. Regarding Rees et al.'s^18^ study, prophylactic ICD therapy in elders was categorized into age groups: 65–60, 65–75, and >75. another study emerged that those over 75 are considered “elders,” and those over 80 are weighted as “very elderly.”^19^ In accordance with the previous result, Scheurlen et al.^7^ classified patients under ICD therapy more than 75 years old as an “elderly” group. It is also pertinent to note that life expectancy may also influence these definitions in different countries; as a result, we considered the predicted life expectancy at birth in Iran in 2010 as the lowest age limit for this study. Therefore, our findings can be valuable compared with those of other societies and countries.

Based on our study results, we found that a low baseline EF is an independent predictor of mortality. This result is closely related to other literature in which low EF, and advanced age, are the most significant predictors of mortality following ICD therapy.^20^, ^21^ We also observed a significantly higher mortality rate among those with severe comorbidities than those without comorbidities. As these comorbidity groups had low numbers of patients, none appeared to be a potential predictor for mortality. Overall, our findings are compatible with previous studies on octogenarian patients in that they all showed a survival benefit of ICD therapy.^10^, ^11^, ^22^

Nonetheless, the primary endpoint of our study was any cause of death, whether related or unrelated to ICD therapy. A multivariable and univariable regression model was used to account for the influence of unrelated causes. In utilizing these models, we sought to neutralize unrelated causes and focus on the specific effects of ICD therapy. However, establishing a definitive relationship between death causes and ICD therapy may require further investigation.

In summary, clinicians should balance the cost and benefit of ICD therapy in elderly candidates and consider the probable complications and potential injury to the myocardium.^23^ While randomized controlled trials may not have been conducted to evaluate survival benefits for ICD in elderly patients‐ likely due to ethical concerns‐ there is an enormous amount of data from NCDR's ICD registry using Medicare claims.^24^ Furthermore, noncardiac comorbidities influence survival in the case of ICD recipients. It is widely noted that noncardiac comorbidities and frailty should be included in evaluating an elderly patient for ICD implantation.^25^, ^26^ Therefore, offering this therapy should be decided individually for every patient. Recently, ESC guidelines suggest that in elderly patients whose a benefit from the ICD is not anticipated due to the patient's age and comorbidities, the omission of ICD implantation for primary prevention may be considered.^8^

### 4.1 Study limitations

Our study has some shortcomings. First, the therapeutic policies of our referral center are based more on secondary prevention, which may cause a selection bias and reduce the generalizability of the study. Although this study was able to demonstrate the safety of ICD in the elderly, but can not demonstrate efficiency because of the lack of a control group. Therefore, a longitudinal study comparing patients with and without ICD therapy after the predicted age of life expectancy can clarify the definite clinical benefit of this treatment in elderly patients and its pure impact on survival. Without a control group in a randomized trial, the utility of ICD therapy cannot be judged. Lastly, the limited number of our study population and the used age life expectancy specific to Iran limit our findings’ generalizability. Thus, drawing decisive conclusions about the benefits and identifying the risks of ICD therapy beyond the age of life expectancy demands further studies.

## 5 CONCLUSION

Aging is accompanied by life‐threatening cardiac conditions, including ventricular tachycardia and heart failure, and thereby ICD therapy could be an effective treatment for elderly patients. However, decision‐making about ICD implantation in elderly patients is not as straightforward as it may seem. The present study shows an acceptable survival benefit in ICD recipients after the predicted age of life expectancy. It underlines the role of baseline EF as the main predictor of mortality. Based on the current knowledge and until obtaining evidence from a clinical trial designed specifically for elderly patients, we should consider predictors of mortality plus the individual characteristic while deciding on ICD therapy in an elderly patient. This information may help physicians better select patients who would most benefit from ICD therapy and improve clinical outcomes in elderly patients.

## AUTHOR CONTRIBUTIONS

**Alireza Malekrah**: Conceptualization; data curation; writing—original draft. **Akbar Shafiee**: Formal analysis; investigation; project administration; writing—review & editing. **Amirhossein Heidari**: Data curation; writing—review & editing. **Ali Vasheghani‐Farahani**: Supervision; writing—review & editing. **Ali Bozorgi**: Project administration; supervision. **Saeed Sadeghian**: Project administration; supervision. **Ahmad Yaminisharif**: Conceptualization; project administration; supervision; visualization; writing—review & editing.

## CONFLICT OF INTEREST STATEMENT

The authors declare no conflict of interest.

## ETHICS STATEMENT

In accordance with our hospital's routine, all patients provided written informed consent upon admission, granting permission for their clinical data to be used anonymously for research purposes. Tehran University of Medical Sciences and the Cardiology Department Research Committee approved the study protocol. (Filing code: 782, Date: 21/12/2015). This study conforms to the ethical principles outlined in the Declaration of Helsinki and its updates.

## TRANSPARENCY STATEMENT

The lead author Ahmad Yaminisharif affirms that this manuscript is an honest, accurate, and transparent account of the study being reported; that no important aspects of the study have been omitted; and that any discrepancies from the study as planned (and, if relevant, registered) have been explained.

## Data Availability

The data underlying this article will be shared on a reasonable request to the corresponding author.
